# Use of large language model (LLM) to enhance content and structure of a school of dentistry LibGuide

**DOI:** 10.5195/jmla.2025.2084

**Published:** 2025-01-14

**Authors:** Emily P. Jones

**Affiliations:** 1 epjones3@email.unc.edu, University of North Carolina at Chapel Hill (UNC) Health Sciences Library, Chapel Hill, NC

**Keywords:** Generative AI, Artificial Intelligence (AI), LibGuides, Large Language Models

## Abstract

A librarian used a large language model (LLM) to revise a dentistry subject LibGuide. Prompts were used to identify methods for optimizing navigational structure for usability, highlight library-specific information students need additional help with, and write summaries of page content. Post-revision, LibGuide access increased, and students provided anecdotal feedback that they perceive the changes positively. LLMs may enhance LibGuide discoverability and usability without adding significant time and resource burdens for librarians.

## BACKGROUND

Large language models (LLMs) like Chat-GPT [1] and Claude.ai [2] are useful tools for summarizing, predicting, and generating text. These tools have potential to increase productivity and decrease the time burden of common, text-based tasks for librarians like LibGuide content creation.

## VIRTUAL PROJECT DESCRIPTION

In June 2024, a librarian used an LLM, Claude.ai, to facilitate a major redesign of a dentistry LibGuide. Through a series of prompts, the librarian consulted the LLM to generate introductions summarizing content of specific pages and to restructure the LibGuide, formerly organized by resource format. Screenshots of the LibGuide pre- and post-revision, as well as examples of prompts provided to, and responses received from, Claude.ai are accessible via the author's institutional repository.

There was a 131% increase in LibGuide access from June - September 2024 (n = 2,288) compared to the same period the year before (n = 989). To the author's knowledge, no other changes were made that would significantly impact usage like new outreach or instruction. In addition to the increase in usage statistics, students have provided anecdotal feedback that they perceive the LibGuide to be more user-friendly and useful after the revision.

## DISCUSSION

LLMs are cost-effective, as most are free, low-cost, or institutionally provided, and time-saving. Large amounts of text can be generated in a matter of seconds, whereas comparable output by a librarian may take hours. Additionally, LLMs can be used across various aspects of medical librarianship across any discipline and can be used to generate or clarify text about complex research topics like systematic reviews and data management.

While there are advantages to using LLMs like increasing efficiency and productivity, there are challenges as well. Concerns have been raised about accuracy of responses, privacy, and algorithm bias [3]. While LLMs are skilled at text-based tasks, they may not be able to adequately produce responses that require nuance, context, or complexity of thought. Therefore, it is best practice to review LLM responses for clarity and accuracy before using them. Additionally, LLM responses are highly dependent upon prompts received. Responses also change each time they're provided, even if the same prompt is provided, whether by the same or different individuals.

## CONCLUSION

This project demonstrates a practical example of how librarians can apply generative artificial intelligence (AI) technologies to routine tasks like LibGuide revision and content creation. Using LLMs to develop page introductions and to reorganize content resulted in a usage increase. While not causative, while not causative, this increase may be correlated to increased discoverability and usability from using generative AI developed text and suggestions. LLMs can enhance the instructional component of LibGuides without adding a significant time burden for the creator.
